# Some of the Drugs Used in Dentistry

**Published:** 1884-07

**Authors:** Charles W. Glassington


THE
AMERICAN JOURNAL
OF
DENTAL SCIENCE.

VOL. XVIII. THIRD SERIES—JULY, 1884. NO. 3.

ARTICLE I.

### SOME OF THE DRUGS USED IN DENTISTRY.

BY CHAS. W. GLASSINGTON, M. R. C. S., L. D. S., EDIN.

(A Paper read before the Student's Society of the National Dental College.)

The subject of my paper this evening is one which is of great value to the Dental Surgeon, and yet during the two, three, or four years a man takes to qualify himself in Dental Surgery, it receives little or no attention from him. This is partly the fault of the student and partly the fault of "the powers that be." A student desirous of obtaining the Diploma of L. D. S., Eng. is required, amongst other things, to attend one course of Materia Medica, and during that course what does he learn to help him in the profession he is ultimately going to follow? Little or nothing at all. He will, if he be diligent enough to attend all the lectures, and attentive when he does attend them, learn that iron, matico, and tannin are hæmostatics, and consequently useful in hæmorrhage after tooth extraction. That arsenic is used for destroying exposed pulps; that carbolic acid and creasote are largely used by dentists; that croton chloral, chloride of ammonium, and tincture of gelsemium are, good for neuralgia; and other important facts. But of the action of these drugs in particular relation to the teeth he gets no information beyond what he acquires during his practice at a dental hospital, or from what he reads in the dental journals, or from the only work (that I know of) on Dental Materia Medica—that by Mr. Stocken. Again, if he reads the text books on Materia Medica, he will find passages like this:—"Externally applied creasote allays toothache depending on caries." Now, although this is true to a certain extent, how very meagre that information is when we know how largely this drug is used by dentists.

During my late appointment as House Surgeon at this hospital, I was on several occasions much impressed by the ignorance shown by some of the students of the different drugs they were using. In dressing "a dead tooth" it was a matter of total indifference to some what they did it with. Eucalyptus, carbolic acid, or creasote, whichever they thought of first, but why they used one or the other they had no definite reason. Now this I maintain is the fault of the "powers that be," and the sooner a chair of Dental Materia Medica is founded at all our dental schools the better it will be for the future of our profession.

I shall endeavor as far as I can to make this paper as practical as possibie, and give you very little theory. I do this for two reasons, viz., that time will not permit me to say much upon each drug; and secondly, it will not make you think that I am giving a lecture.

The first drug I will put before you is ARSENIC, and is one on which alone anybody could write an interesting paper for discussion. Arsenic is an irritant, escharotic, and powerful antiseptic, the latter fact being proved by the injection of bodies for dissection. I do not intend starting the question whether arsenic should or should not be used for destroying pulps, although, for my own part, I am quite of opinion that when a pulp or part of one can be saved it is our duty to save it. We all know that to preserve the life of a tooth we must preserve the life of the pulp and of the peri-dental membrane, and in using arsenic we destroy one if not both. Broadly speaking, the only indication I shall put down for its use, is when the whole of the pulp is inflamed and pus has formed.

Assuming we have decided to destroy a pulp with arsenic, let me impress on those who are just commencing their hospital work the following hints:—

1. Apply the rubber dam.

2. Carefully dry the cavity first with cotton wool or bibulous paper, and afterwards with the hot air syringe.

3. If the exposure is small enlarge it with sharp excavators, as arsenic applied to a small exposure only increases the inflammation and pain.

4. Apply the arsenic (about the 1-16 gr.) on a piece of cardboard sufficiently large to prevent any pressure on the pulp, and soaked either in carbolic acid or creasote, cover with copal varnish, and fill with Hill's gutta-percha. I prefer this to mastiche, as you are liable to drag the mastiche away when taking off the rubber; besides which it is a safer filling. Whatever fluid you use to convey the arsenic to the cavity, be careful not to have an excess, as you are liable to have this excess squeezed out and with it some of the arsenic.

5. Do not use it in young children unless you know the tooth is fully formed, for in destroying the pulp of a growing tooth you stop its growth.

6. Should a pulp be greatly inflamed use an application of creasote, tannin, and morphia, to reduce the inflammation before using the arsenic.

7. Do not use it in excess as the effects spread to periosteum, bone, and neighboring pulps, and may cause their destruction.

Mr. W. E. Harding brought a case before the Odontological Society on December 5th, 1881, in which he had carefully treated a pulp with arsenic, and in three days the patient had all the symptoms of arsenical poisoning.

Mr. Coleman has lately brought before the notice of the profession a method of treating so-called " dead teeth " with arsenic. In those cases where the pulp has sloughed away, the canal cavities filled with puriform fluids, the dentine itself being moist and very offensive, and as a rule, accompanied with periodontal irritation, due to the presence of septic substances, he has used arsenic with better results than ten or twenty applications of carbolic acid or creasote. His mode of procedure is this:—He simply clears out the pulp cavity, washes with carbolic acid, and applies arsenic precisely as if for destruction of a pulp, laying the application over the orifice or orifices of the fang cavities, and then filling over with oxycloride of zinc. After an interval of two or three months, if the filling is removed, the pulp and fang cavities will be found dry and perfectly sweet. This method of treating teeth is one which I should not feel inclined to differ from, coming as it does from such an authority; but the fact is I had myself partly done the same thing in two or three cases, before I was aware Mr. Coleman was pursuing that course of treatment. But in the few cases I tried it, it was more from necessity, and because I did not know what else to do. I well remember two cases, both were upper molars, distal cavities with suppurating pulps. The patients, who were going into the country, refused to have them extracted, and wanted them stopped. So after applying the rubber dam and carefully excavating, I placed a large cap of cardboard, on one side of which was a paste of creasote, morphia, and arsenic, over the pulp cavity, filled with osteo, and told the patient that in the event of any pain or swelling to go and have the tooth out. Both cases did well, and one came back a short time ago to have the tooth filled up again, the osteo having worn down. The remains of the filling still covered the pulp cavity, so I simply put in another filling as the patient begged me not to disturb it, it being so comfortable. My experiences are not sufficient to enable me to speak well of this mode of treatment; but when a gentleman like Mr. Coleman brings forward any addition to our knowledge of Dental Surgery, we may be sure he has good reasons for doing so.

Arsenic is also useful for allaying the pain of sensitive dentine in *shallow cavities*. Of the chemical and physiological action of this drug I must refer you to Dr. Arkovey's experiments, which are published in *Transactions of the Medical Congress*, and also in the *Dental Record* for February 1882.

The next drug I shall speak of is CARBOLIC ACID. This is a powerful antiseptic, caust c and escharotic. In Dentistry it is used chiefly in a diluted strength as in antiseptic for dressing the canals of teeth. Strong Carbolic is also useful for allaying the pain of Sensitive Dentine, which it does by coagulating the contents and sealing-up the ends of the dentinal tubes ; and by applying it to the gum after extraction it deadens sensibility. When used for capping exposed pulps it should not be stronger then from 1 to 20, as a solution of greater strength acts as an irritant, destroys the layer of odontoblasts, and so probably prevents the normal formation of secondary dentine.

In combination with Glyscerine about 1–12, it may be used to increase the secretion of the palate for the purpose of retaining suction plates, which it does by stimulating the action of the mucous-follicles. Of its value in the treatment of alveolar abscess I cannot speak, preferring another drug I shall mention later on. It is very useful as a lotion, when diluted, for nlcerations of the mouth, and with Glycerine (Scruples ss. to Drachm 1 of Glyscerine, and diluted with about 4 Scr. to 6 Drams of water) is good in cases of sore throat with relaxed uvula and soft palate. In cases after the removal of tartar the edges of the inflamed gums are greatly improved by the application of Carbolic Acid. Should any of you be unfortunate enought when using it to allow any to come into contact with the cheeks or lips of your patient, its injurious effects are greatly counteracted by anointing the parts with olive oil.

CREASOTE is a drug I scarcely use by itself. It is a powerful deodoriser, antiputrescent, and antiseptic, and acts locally as a caustic by coagulating albumen in solution

The chief use I have put it to is in cases of caries of the temporary teeth which are painful, and which require stopping to prevent their too early removal. It is said to have great effect in the tooth-ache of pregnancy.

The late Dr. Dean, of Chicago, said that for alveolar abscesses with fistulous opening on the gum, Creasote soon effects a cure ; but in those cases where there is no opening, neither that nor Carbolic Acid should be used, as these drugs coagulate the albuminous ingredients of the pus, and effect its fluidity and thus increase the difficulty of drawing it through the apical foramen ; but after the contents of the sac are got rid of, then Creasote may be used with advantage.

EUCALYPTUS.—This drug is one in which I have the greatest faith as an antiseptic. It possesses none of the irritating properties of Carbolic Acid or Creasote, and is not a caustic. It is highly destructive to all low forms of organic growth ; and, according to some, is three times as strong as Carbolic Acid in preventing the formation of bacteria. Professor Lister says, "That while entirely free from any toxic or locally irritant effects it is a perfectly trustworthy antiseptic." On the other hand, Dr. Schlemitz, of Arnsberg, says it is not a powerful antiseptic. Mr. Stocken, in his work on *Dental Materia Medica*, says, "apart from its antiseptic properties it is said to be a local anæsthetic, lulling the sensibility of tooth-ache and sensitive dentine." I have used it with great success in all cases of chronic alveolar abscess in the following manner:— I first of all apply the rubber dam, clear away any particles of food or softened dentine, and then bathe the cavity with Eucalyptus. I then proceed to open up the canals using, as far as possible, all antiseptic precautions, for example, bathing the cavity with the oil, using instruments the points of which have been dipped in the oil, and then when I have opened the canals, pumping up more Eucalyptus on a Donaldson's probe. If the tooth is not very tender, and the patient can bear it, I shape the cavity the same day ; but, if not, I pump up more Eucalyptus, the antiseptic and antiputrescent qualities of which are greatly increased if used with Iodoform, either in solution, or by carrying it up the canal on some cotton-wool soaked in Eucalyptus, and seal the cavity with Hill's gutta-percha. Again, I prefer gutta-percha to mastiche, as in these cases it not only keeps the cavity much cleaner, but the fluids of the mouth have less power of permeating through the filling, and thus preventing the introduction of any fresh germs. When I next see my patient—say in two days—I again apply the rubber dam, remove the temporary filling, and if the canals are tolerable sweet and there is no sign of pus formation, I shape the cavity, providing I have not done it before, pump up more Eucalyptus, take a small piece of cotton-wool soaked in it and force it up to the end of the canal, so that it shall occupy only a small portion of the end of the canal. I then take some small pieces of Hill's gutta-percha, dipped in the oil, and fill the remaining portion of the canal, and on the top of this put either gutta-percha or one of the osteo fillings, and direct my patient to come and see me at the end of six months to have a permanent filling inserted.

Those cases which I have seen at the end of that time, have all been successful. I ought to mention that in some cases it requires more than the second visit before you can put in the temporary filling; for instance, at times I have noticed when taking out the first dressing you will find a small bead of pus come down. In a case like this a third or even fourth visit will be necessary before the tooth can be stopped.

IODOFORM possesses strong antiseptic powers, and is also slightly anæsthetic and sedative in its action. I have seen it used in this hospital in cases of chronic alveolar abscess, but success with it was not so great as I anticipated unless used with Eucalyptus. This drug has the credit of not possessing any irritating properties, but Dr. Phillips, late lecturer on *Materia Medica* and *Therapeutics*, at Westminister Hospital, to whom I am greatly indebted, in more ways than one, for my knowledge of the inorganic part of the *Materia Medica*, says:—"In certain cases Iodoform is an irritant, viz., when applied to abraded surfaces, especially inflamed ulcers."

The great objection to its use is its odor, but this to a certain extent may be covered with one of the following drugs—Balsam of Peru, Eucalyptus, Otto of Rose, or Tannic Acid; but none of these cover it entirely—Balsam of Peru being the best.

IODINE and ACONITE.— I shall speak of these two drugs chiefly in combination as they are generally so used by dentists.

The following hints I venture to hope will not prove unacceptable:—When applying them to the gum always keep the fluids of the mouth away from the part painted till the Alcohol has sufficiently evaporated, or else the application will be washed away. The part always assumes a metallic lustre when the Alcohol has evaporated.

Be carefel when using it not to have an excess of fluid on the cotton-wool or brush, for fear any of the superfluous fluids should find its way down the throat, as very small doses of Aconite have proved fatal.

In the event of any serious effects happening to you, the following are some of the antidotes—Animal Charcoal, an emetic of Sulphate of Zinc (do not be afraid of giving too much,) Brandy and Ammonia internally; stimulation externally, subcutaneous injection of Atropine.

Should you prescribe Aconite as an external remedy for Neuralgia, warn the patient to keep the eyes closed when using it; or extensive irritation may be set up in the eye.

Do not forget that Fleming's Tincture of Aconite is five or six times as strong as that of the B. P. Iodine by itself is used in cases of Ranula, by swabbing out the cyst after it has been opened and its contents evacuated. For my part, I much prefer to stuff the cavity with lint and so set up inflammation and granulation. In Gingivitis it is good after the removal of Tartar, for touching-up the edges of the gum; but as I pointed out before I use Carbolic Acid instead.

Locally applied it acts as a stimulant, escharotic and absorbent. It is useful in all forms of Periodontitis as it promotes absorption of all abnormal material by stimulating the absorbents to increased action, promoting and hastening healthy action ; and as a counter irritant by directing the circulation to another part.

IRON.—In speaking of this drug I shall confine myself only to its action as a hæmostatic. For the last eight years I have not used anything else but Liquor Ferri Perchloridum to arrest hæmorrhage after tooth extraction, and have never found it fail ; consequently, I am in a position to speak of its value.

My mode of procedure is simply this :—As soon as I see the patient I mop out the cavity, clear away all clots, make the patient wash the mouth out well with cold water, and plug with a plain piece of cotton-wool. I then take another piece of wool soaked in the Liquor Ferri Perchlor., remove the first piece, and plug the socket tightly, placing a good pad on the top of this, and tell the patient to keep the teeth together. If necessary I bind up the jaw.

Then just a few precautions to the patient, such as keeping quiet, keeping in an upright position, and taking everything cold.

I use the strong solution of the perchloride in preference to the tincture, as its astringent effects are greater, but it is more acid and often acts as an irritant, consequently in certain cases it may be used with a little water or glycerine.

Its great objection to surgeons using it is that it prevents union by first intention. Iron acts as a true styptic on the blood; it causes a coagulum to form—it narrows both arteries and veins at the place of application, whilst the neighbouring vessels become dilated. Its action varies, however, according to the strength and kind of the preparation used.

ALUM acts as a simple astringent by contracting the arteries and muscular fibres of the part touched by it, and renders the surface pale and dry. If there is not sufficient fluid present to saturate the Alum, it affects the deeper tissues and acts as a caustic. Most astringents favor the action of Alum, but as Tannin decomposes it, they should not be used together, as they may prove less astringent.

It is useful in Simple Stomatitis; but in Ulcerative Stomatitis Chlorate of Potash is a specific. As a hæmostatic I never employ it; but care should be taken not to use too strong, especially in scrofulous patients, as it may cause unhealthy ulceration.

TANNIC ACID is a most powerful external astringent, and there are many before me this evening who use it in preference to any other drug. Applied externally it is preferable to Gallic Acid, but for an internal astringent Gallic Acid should be given, as Tannic Acid becomes converted in the blood into Gallic Acid and grape sugar, and therefor; only part of it is available. For spongy gums it is a useful application, and combined with Creasote it is frequently used in ulceration of the pulp.

MATICO, either in leaf or powder, is an effective styptice but its power is supposed to be due to the mechanical structure of the leaf which, when applied, should have the underside placed next to the bleeding surface.

Chloride of Ammonium, Tincture of Gelsemium and Croton Chloral are used by the Dental Surgeon for the relief of Neuralgia.

I use these drugs as follows:—A patient comes to me suffering from neuralgia, I at once prescribe Ammonii Chloridum gr. XXX, Tr. Limonis Dr. ss. (to cover the nauseous taste of Amm. Chlor.,) Sp. Chloroformi m xv., Aquæ rd Sc. j., every three hours. Should this not give relief in three doses it should be discontinued; and then I give Croton Chloral gr. iij. to X, Sy. Solu, Dr. j., Aquæ ad Sc. j., and I have never found one of these too fail me. Of the Tincture of Gelsemium I have no knowledge beyond what I have read and heard from others; but its value as a sedative in neuralgia is, I believe, very great.

BROMIDE OF POTASSIUM.—This drug is not much used by Dentists, but I mention it to show its great value in convulsions due to teething. Dr. Phillip's in his work on *Materia Medica*, says, "In the restlessness and nerve irritation on convulsions, sometimes attendant on Dentition, bromides are exceedingly useful, so that the gum lancet is scarcely ever needed." In a child, say 12 months old, with convulsions, I give Pot. Brom. gr. iij., Sy. Simp. m X, Aq. Anethi. ad Dr. j., every three or four hours, at the same time I order a small dose of Calomel as a purge, hot baths, and ice to the head.

Chlorate of Potash is another valuable drug to the Dentist in all forms of inflammation of the mouth. In Ulcerative Stomatitis given both internally and as a gargle it acts as a specific, and the same must be said of it in Tonsilitis, particularly when combined with the Tincture of the Perchloride of Iron.

The last drug I shall speak of is the Permanganate of Potash. This I conceive to be one of the most valuable medicines in *Dental Materia Medica*, I use it for two things, first for testing whether in a carious tooth the offensive smell comes from a suppurating pulp or from the gum.

Its other great use is when a patient comes with a mouth full of suppurating pulps and polypi. The odor from these is simply unbearable, but with a gargle of Pot. Perman, the Dentist is enabled to bend over his patient with a certain amount of comfort.

In conclusion, I must thank you for the very patient hearing you have given me during a somewhat lengthy paper. I could not go so deeply into the action of some of the drugs as I should have liked, on account of the time, and some I have had to leave out all together. I feel quite sure that lectures on *Dental Materia Medica* and *Therapeutics* at our Dental Schools are greatly needed. Should this ever come to pass the dental student would not only have the opportunity of turning out a better Dentist; but would be able during his student's career to use and judge of those drugs which, under the present system, he seems to have such a very vague idea.—*London Dental Record.*

